# The effect of thioredoxin‐1 in a rat model of traumatic brain injury depending on diurnal variation

**DOI:** 10.1002/brb3.3031

**Published:** 2023-05-08

**Authors:** Roxana Noriega‐Navarro, Ricardo Jesús Martínez‐Tapia, Rubén González‐Rivera, Alicia Ochoa‐Sánchez, Julio César Abarca‐Magaña, Lucía Landa‐Navarro, Verónica Rodríguez‐Mata, Perla Ugalde‐Muñiz, Armando Pérez‐Torres, Abraham Landa, Luz Navarro

**Affiliations:** ^1^ Departamento de Fisiología, Facultad de Medicina Universidad Nacional Autónoma de México Ciudad de México México; ^2^ Departamento de Microbiología y Parasitología, Facultad de Medicina Universidad Nacional Autónoma de México Ciudad de México México; ^3^ Simons Initiative for the Developing Brain, Centre for Discovery Brain Sciences University of Edinburgh Edinburgh UK; ^4^ Departamento de Biología Celular y Tisular, Facultad de Medicina Universidad Nacional Autónoma de México Ciudad de México México

**Keywords:** diurnal variation, neuroprotection, thioredoxin, traumatic brain injury

## Abstract

**Introduction:**

Traumatic brain injury (TBI) is a public health concern with limited treatment options because it causes a cascade of side effects that are the leading cause of hospital death. Thioredoxin is an enzyme with neuroprotective properties such as antioxidant, antiapoptotic, immune response modulator, and neurogenic, among others; it has been considered a therapeutic target for treating many disorders.

**Methods:**

The controlled cortical impact (CCI) model was used to assess the effect of recombinant human thioredoxin 1 (rhTrx1) (1 μg/2 μL, intracortical) on rats subjected to TBI at two different times of the light‐dark cycle (01:00 and 13:00 h). We analyzed the food intake, body weight loss, motor coordination, pain perception, and histology in specific hippocampus (CA1, CA2, CA3, and Dental Gyrus) and striatum (caudate‐putamen) areas.

**Results:**

Body weight loss, reduced food intake, spontaneous pain, motor impairment, and neuronal damage in specific hippocampus and striatum regions are more evident in rats subjected to TBI in the light phase than in the dark phase of the cycle and in groups that did not receive rhTrx1 or minocycline (as positive control). Three days after TBI, there is a recovery in body weight, food intake, motor impairment, and pain, which is more pronounced in the rats subjected to TBI at the dark phase of the cycle and those that received rhTrx1 or minocycline.

**Conclusions:**

Knowing the time of day a TBI occurs in connection to the neuroprotective mechanisms of the immune response in diurnal variation and the usage of the Trx1 protein might have a beneficial therapeutic impact in promoting quick recovery after a TBI.

## Graphical abstract

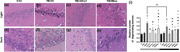

## 1 INTRODUCTION

Traumatic brain injury (TBI) is defined by Menon et al. (2010) as an alteration in brain function or other evidence of brain pathology caused by an external force (Menon et al., 2010). Post‐TBI, two types of brain damage are generated: the primary lesion that occurs at the moment of impact and is considered irreversible and the secondary lesion that is developed over a period of hours to days and exacerbates the damage of the primary lesion, constituting the main cause of death reported by hospitals. Several processes occur during secondary injury, such as neurotransmitter release, free radical formation, calcium‐mediated damage, gene activation, mitochondrial failure, and inflammatory responses (Mass et al., 2008).

Thioredoxin (Trx) is ubiquitously present in all organisms, and it is part of the thioredoxin system (TrxS), which consists of an electron donor (NADPH) and other antioxidant oxidoreductase protein (thioredoxin reductase, TrxR). Human cells contain three types of thioredoxins: cytoplasmic Trx1, mitochondrial Trx2, and spermatozoa Trx3 (Silva‐Adaya et al., 2014). Trx1 is a small 12 kD protein with an active conserved site, Cys‐Pro‐Gly‐Cys, which is essential for its function as both an active oxidoreductase and an electron donor for some peroxiredoxins (Lu & Holmgren, 2014; Silva‐Adaya et al., 2014). This protein engages in various physiological tasks due to its redox characteristics including antioxidants, gene transcription, DNA synthesis and repair, cell signaling, cell cycle, inflammation, and apoptosis (Mahmood et al., 2013). Previous studies have reported that induction or overexpression of Trx1 contributes to the brain's tolerance and neuroprotection against insults. For example, pretreatment with estradiol benzoate reduces ferrous citrate‐induced brain injury in female rats increasing brain Trx activity (Chen et al., 2010); overexpression or intravenous administration of recombinant thioredoxin (rTrx1) decreased ischemic neuronal injury in the murine MCAO model (Hattori et al., 2004; Takagi et al., 1999).

Numerous studies indicate that: (1) there is a close correlation between the immune system and the circadian system; (2) the time of day is critical to the nature of the immune response (innate and adaptive); and (3) their dysregulation may lead to inflammatory diseases or immunodeficiency (Curtis et al., 2014; Labrecque & Cermakian, 2015; Martínez‐Tapia et al., 2020a). We have previously reported that recovery from TBI is better in rats if the injury occurs during the dark phase of the diurnal cycle (Estrada‐Rojo et al., 2018b; Martínez‐Tapia et al., 2020b; Martinez‐Vargas et al., 2006, 2013). Therefore, the present study aims to assess, at the behavioral and histological levels, the neuroprotective role of Trx1 in a rat model of TBI and if this presents a diurnal variation. Additionally, the pain response was evaluated by the rat grimace scale (RGS).

## 2 MATERIALS AND METHODS

### 2.1 Animals

Adult male Wistar rats of 9–10 weeks of age (250–300 g) were housed for 21 days at a constant temperature (21–23°C) from our breeding facilities. They had access to food and water ad libitum in a 12:12 light cycle (lights on from 07:00 to 19:00) or dark cycle (lights on from 19:00 to 07:00). The food intake and body weight of the animals were recorded every 2 days before and 24, 48, and 72 h post‐TBI. All experiments followed the recommendations of the Ethics Committee of the Faculty of Medicine, Universidad Nacional Autónoma de México (UNAM) (project 018/2016 approved April 05, 2016), the Animal Care and Use Committee, and the Mexican Official Regulations (NOM 062‐ZOO‐1999). All efforts were made to reduce the number of animals and their suffering.

### 2.2 Drugs

Recombinant human thioredoxin (rhTrx1) was obtained from Abcam (Cambridge, MA), and minocycline (Mino) was purchased from Sigma‐Aldrich (St. Louis, MO). Both drugs were dissolved in 0.9% NaCl (sterile saline [SS]), which was used as a vehicle.

### 2.3 Brain injury by the CCI model

Moderate Controlled Cortical Impact (mCCI) (Dean et al., 2018) was produced under deep anesthesia with a mixture of ketamine, xylazine, and acepromazine (66, 0.26, 1.3 mg/kg). The rat was positioned in a stereotaxic frame to fix the skull. Using aseptic techniques, a midline scalp incision was performed, the skin and fascia were removed to expose the skull, and a craniotomy (6 mm) was made centered midway between the lambda and bregma to expose the dura without damaging the meninges or the cortex. A cortical contusion was produced using a calibrated pneumatic piston device (Martinez‐Vargas et al., 2013). mCCI was produced with a 5‐mm diameter impactor tip that compressed the cortex to a depth of 2.5 mm at 50 psi of pressure. The incision was sutured closed. Sham rats received the same surgical procedure without the cortical impact.

### 2.4 Experimental design

Rats were administered intracortical in the motor cortex (AP = −2, *L* = +1.5, *H* = −2; Paxinos & Watson, 1998), with rhTrx1 (1 μg/2 μL) or minocycline (160 μg/4 μL, Gong et al., 2018) as a positive control or 4 μL of SS as the vehicle, at 0.5 μL/min, 15 min after a TBI or sham surgical procedure. The dose of hTrx1 was selected based on pilot studies in the laboratory. Rats were divided into seven groups for each phase of the cycle (light or dark): (1) Naïve (N), (2) Sham with SS (S/SS), (3) TBI with SS (TBI/SS), (4) Sham with rhTrx1 (S/rhTrx), (5) TBI with rhTrx1 (TBI/rhTrx), (6) Sham with minocycline (S/Mino); and (7) TBI with minocycline (TBI/Mino). Experiments were done at 13:00 h for light phase groups or at 01:00 h for dark phase groups.

### 2.5 Behavioral analysis

#### 2.5.1 Balance beam (distance)

Motor coordination deficits were measured by the number of missteps crossing a square wooden beam (1 m long by 2 cm) or a circular wooden beam (1 m long by 2 cm in diameter). The beam was elevated 40 cm above the table on wooden supports to perform the test. A 12 × 15 × 26 cm black escape box was placed at the opposite end with a 7 × 9 cm opening (Radabaugh et al., 2016). Three days before TBI, all animals were pretrained to encourage reliable crossing. Rats could walk across the beams five times to traverse it without reversing or stopping rapidly. Animals were assessed on the test beam before and 24 and 72 h post‐TBI.

### 2.6 Pain analysis

The animals were videotaped for 5 min before and 24 and 72 h post‐TBI, in a quiet environment, with white light, without experimenters. Scoring was conducted according to the method described by Sotocinal et al. (2011). The captured face images in Portable Network Graphic format (10 images/rat) were copied into PowerPoint 2013 (Microsoft Com), one image per slide. Images were presented in random order to scorers. Three trained scorers, blind to the treatment allocation, rated four separate action units in each image: orbital tightening (eye), nose and cheek flattening, ear change, and whisker change. Each action unit in each image was rated using a 3‐point scale (0 = not present, 1 = moderately present, 2 = obviously present). The average rating for the four action units was determined first. Subsequently, the average of the three scores was calculated and used as the RGS score for each image. Finally, the average of the RGS scores of 10 images from an individual rat was calculated and used as the RGS score for that rat (Sotocinal et al., 2011).

### 2.7 Histological analysis

Four rats of each group were killed 72 h after TBI. All the rats were deeply anesthetized with sodium pentobarbital (100 mg/kg ip) and perfused transcardially with 200 mL of 0.9% saline phosphate buffer (pH = 7.4) followed by 200 mL of 4% paraformaldehyde in 0.1 M phosphate buffer (pH = 7.4). The brains were removed, and postfixed overnight in paraformaldehyde fixative at 4°C, and then were dehydrated in increasing ethanol concentrations overnight, and embedded in paraffin wax. Sections (4 mm thick) were taken using a microtome and stained with hematoxylin and eosin (H&E). Morphological changes were analyzed according to the method described by Martínez‐Tapia et al. (2020b) in four different areas: three subregions of the hippocampus [CA1, CA2/3, and dentate gyrus (DG)], and dorsal striatum [caudate‐putamen]. Images were acquired using a CX31 Olympus microscope with a digital camera and analyzed with ImageJ software (NIH, MD, USA) (Martínez‐Tapia et al., 2020b).

### 2.8 Data analysis and statistics

Data are presented as the mean ± SEM of independent animals. Statistical differences between groups were determined by one‐ or two‐way ANOVA followed by the Tukey test or with Kruskal–Wallis followed by the Dunn test for variables that do not have a standard distribution. *p* < .05 was considered statistically significant.

## 3 RESULTS

### 3.1 Trx1 improves the recovery of rats subjected to TBI depending on diurnal variation

#### 3.1.1 Food intake and body weight

The neuroprotective role of hTrx1 (1 μg/rat) in a rat mCCI model of TBI at two different points of the light‐dark cycle was assessed by recording food intake and body weight. Behavioral response assessment was performed using a balance beam, and pain response was measured with an RGS score.

**FIGURE 1:**
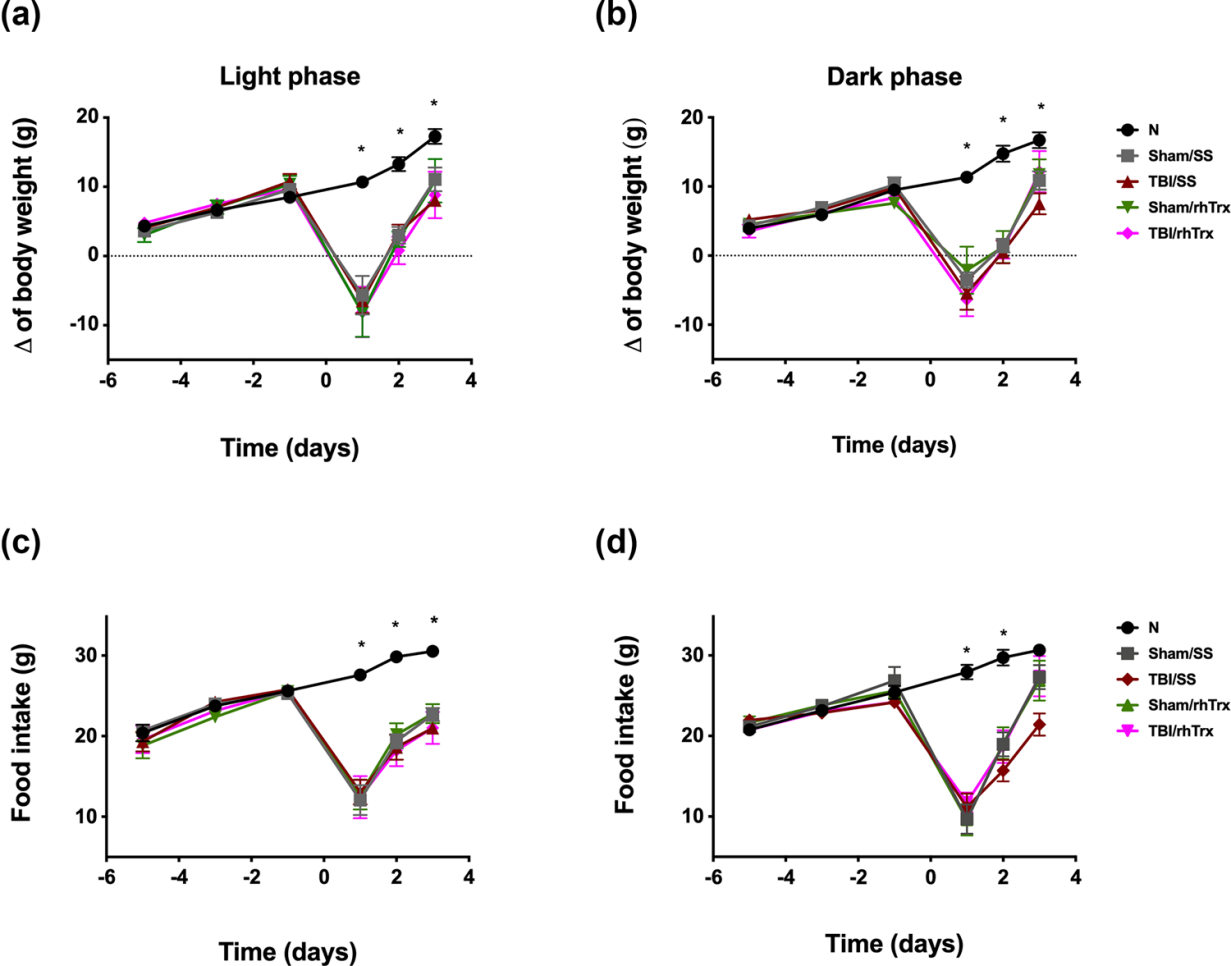
**The effect of rhTrx1 on food intake and body weight after TBI**. Body weight change (a, b) and food intake (c, d) were observed at 24, 48, and 72 h post‐TBI. Data are expressed as mean ± SEM (n = 12). ANOVA and Tukey test as *post‐hoc*, *p <.05 *versus* Naïve (N). Sham (S) or TBI groups were administered with sterile saline (SS) or thioredoxin (rhTrx).

Figure 1 shows the experimental subjects' food intake and body weight change records before, 24, 48, and 72 h post‐TBI/rhTrx. Food intake and body weight decreased after surgery, but they recovered 3 days after TBI. We only observed statistically significant differences between naïve and the other groups 24, 48, and 72 h after TBI. The decrease in food intake and body weight change is more evident in the subjects undergoing TBI during the light phase than the dark phase of the cycle and in the TBI groups administered with the saline solution than in those administered with rhTrx1.

#### 3.1.2 Balance square beam

**FIGURE 2:**
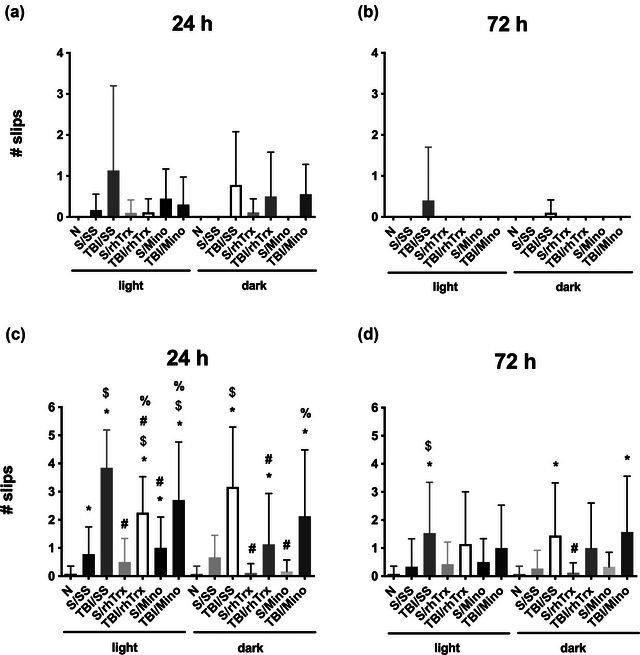
**The effect of hTrx1 and minocycline in gross and fine motor coordination after TBI**. Deficits were assessed as the number of errors or slips in the square (a, b) or round (c, d) beam post‐TBI at 24 and 72 h with rhTrx1 or minocycline. Data are expressed as mean ± SEM (n = 12). Kruskal–Wallis and Dunn's test as *post‐hoc*, *p < .05 versus Naïve (N), ^$^ *p* < .05 versus Sham (S), ^#^ *p* < .05 versus TBI/SS, ^%^ *p* < .05 versus S/rhTrx1. Groups S or TBI were administered with sterile saline (SS), thioredoxin (rhTrx), or minocycline (Mino).

Our data did not show statistically significant differences. However, at 24 h post‐TBI (except for Naïve, S/SS, and S/Mino –in the dark phase of the cycle), all groups had errors in crossing the beam, which were more frequent in subjects with TBI (Figure 2a). At 72 h, only TBI/SS groups exhibited difficulties in performing the task, which were more evident in the light phase of the cycle (Figure 2b). These data suggest that subjects show motor recovery 3 days post‐TBI, especially when administered with rhTrx1 or minocycline, and that TBI was applied during dark hours.

#### 3.1.3 Balance round beam

All groups showed difficulties crossing the round beam, making more mistakes when TBI was applied in the light phase of the cycle. At 24 h post‐TBI, the Naïve group significantly differed from all the other groups during the light phase. The S/SS group presented significant differences with TBI/SS (both phases) and TBI/rhTrx and TBI/Mino (only light phase) groups. rhTrx administration reduced the number of errors in both phases, while Mino did not show an effect (Figure 2c). At 72 h post‐TBI, the Naïve group presented significant differences with TBI/SS (both phases) and TBI/Mino (dark phase) groups. The TBI/rhTrx groups did not show significant differences with TBI/SS groups in any of the phases; however, they were also not different from the Naïve groups. (Figure 2d).

This finer test confirms that experimental subjects showed motor recovery after 3 days post‐TBI, which is more evident in rats administered with rhTrx1.

#### 3.1.4 Pain analysis

**FIGURE 3:**
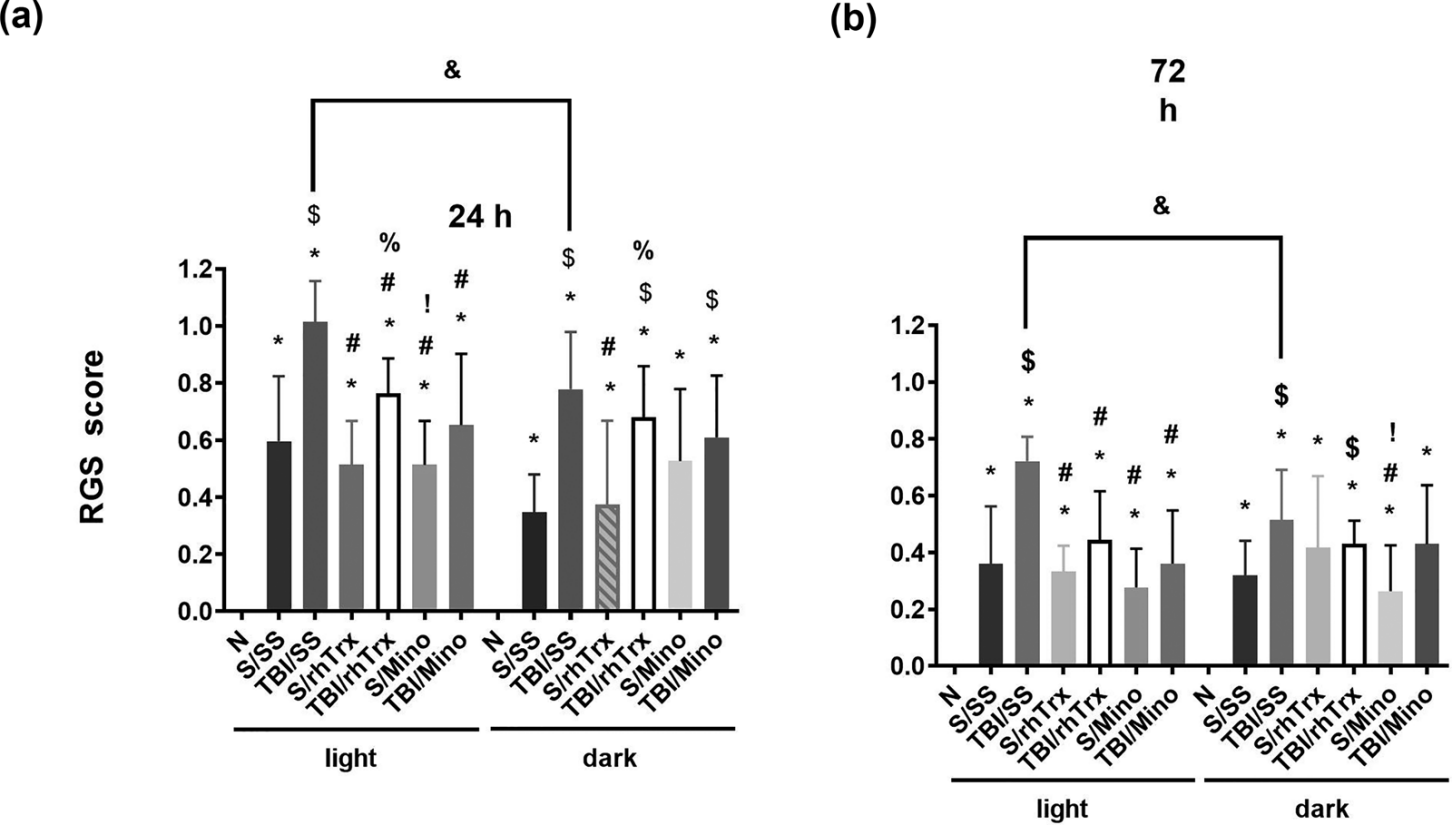
**The effect of rhTrx1 and minocycline on rat grimace scale**. Score observed post‐TBI at 24 h (a) and 72 h (b) with rhTrx1 or minocycline. Data are expressed as mean ± SEM (n = 6). Kruskal–Wallis and Dunn´s test as *post‐hoc*, *p < .05 versus Naïve (N), ^$^ *p* < .05 versus Sham (S), ^#^ *p* < .05 versus TBI/SS, ^%^ *p* < .05 versus S/rhTrx1, ^!^ *p* < .05 versus TBI/rhTrx, ^&^ *p* < .05 between TBI/SS light and dark phases. Groups S or TBI were administered with sterile saline (SS), thioredoxin (rhTrx), or minocycline (Mino).

Except for the TBI/SS group in the light phase of the cycle, the animals presented mean pain responses of less than 1 in the RGS score, which even decreased 72 h after injury. Sham groups showed lower pain scores than TBI groups. However, experimental subjects administered with rhTrx and minocycline had lower pain scores than TBI/SS groups. At 24 h post‐TBI, the Naïve group differed significantly from all experimental subjects. S/SS group presented significant differences with the TBI/SS (both phases) and TBI/rhTrx and TBI/Mino (dark phase) groups. TBI/SS group showed significant differences with the Sham group administered with hTrx1 (both phases), minocycline, TBI/rhTrx, and TBI/Mino (light phase) groups. S/rhTrx significantly differed from TBI/rhTrx (both phases) and TBI/rhTrx from S/Mino (light phase). Significant differences were observed between TBI/SS groups of light versus dark phases (Figure 3a). At 72 h post‐TBI, the Naïve group differed significantly from all groups. S/SS presented differences with TBI/SS (both phases) and TBI/rhTrx (dark phase) groups. TBI/SS group showed significant differences with S/Mino (both phases), S/rhTrx, TBI/rhTrx, and TBI/Mino (light phase) groups, and TBI/rhTrx with S/Mino (dark phase). Significant differences were also observed between TBI/SS groups of light *v*ersus dark phases (Figure 3b). These results indicate that experimental subjects present a moderate pain response according to the mCCI model and suggest less damage in animals subjected to TBI during the dark phase of the cycle, which recovered after 3 days post‐TBI, principally when the rats were administered with rhTrx1 or minocycline. Besides, the results of the RGS score are related to those observed with behavioral analysis.

### 3.2 Trx1 improved neuronal damage in specific hippocampal and striatum regions of rats subjected to TBI depending on diurnal variation

To study the effect of Trx1 on the histopathological extent of secondary brain damage after TBI, we performed morphometric analysis of degenerating neurons in four different hippocampal–*Cornus Ammonis* 1, 2, 3 (CA1, 2, and 3) and DG‐ and striatum‐caudate/putamen‐subregions. At 72 h post‐TBI, the damage in both regions was considered significant in TBI/SS groups, which is more evident if the injury occurs in the light phase of the cycle.

**FIGURE 4:**
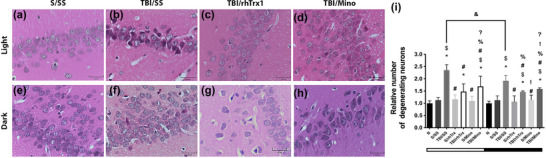
**The effect of rhTrx1 and minocycline on histopathology of the hippocampal subregion CA1**. Figures (a‐f) represent H&E staining of the hippocampal CA1 region in S/SS‐light phase (a), TBI/SS‐light phase (b), TBI/rhTrx1‐light phase (c), TBI/Mino‐light phase (d), S/SS‐dark phase (e), TBI/SS‐dark phase (f), TBI/rhTrx1‐dark phase (g), TBI/Mino‐dark phase (h) phase of the cycle, post‐TBI at 72 h. Relative number of degenerating neurons observed post‐TBI at 72 h with rhTrx1 and minocycline (i). In Light TBI/SS, we evidenced in the pyramidal cell layer (PCL) of CA1 a process known as “acute eosinophilic neuron degeneration.” These red dead neurons (degenerating neurons) are characterized by cell body shrinkage, loss of Nissl substance, intensely stained eosinophilic cytoplasm, and a small/shrunken darkly stained (pyknotic) nucleus. We also note the increased vascular lumen in the region (b). In Light TBI/rhTrx1, we did not observe the presence of red neurons (c), but we did in Light TBI/Mino, although in smaller numbers (d). As for Dark TBI/SS, the lower number of network neurons in the PCL is notable (f). In TBI/rhTrx1, the presence of red neurons is lower, but we observed a larger caliber of the vascular lumen. Finally, in Dark TBI/Mino, the PCL is preserved but with a change in the staining tonality suggesting a basophilic change (also known as dark neurons). Data are expressed as mean ± SEM (n = 4). Kruskal–Wallis and Dunn´s test as *post‐hoc*, *p < .05 versus Naïve (N), ^$^ *p* < .05 versus Sham (S), ^#^ *p* < .05 versus TBI/SS, ^%^ *p* < .05 versus S/rhTrx1, ^!^ *p* < .05 versus TBI/rhTrx, ^?^ *p* < .05 versus S/Mino, TBI/rhTrx, ^&^ *p* < .05 between TBI/SS light and dark phases. Groups S or TBI were administered with sterile saline (SS), thioredoxin (rhTrx), or minocycline (Mino) (bars, 25 μm).

In the hippocampal CA1 (Figure 4), the relative number of degenerating neurons in the Näive group significantly differed from all TBI groups. The S/SS group presented significant differences from TBI/SS, TBI/Mino (both phases), and TBI/rhTrx (dark phase) groups. TBI/SS group showed significant differences from all groups. The S/rhTrx group significantly differed from TBI/Mino (both phases) and TBI/rhTrx (dark phases) groups. TBI/rhTrx group presented significant differences from S and TBI groups administered with Mino (dark phase). The S/Mino group showed only significant differences from TBI/Mino (both phases) group. Significant differences were observed between TBI/SS groups of light versus dark phases.

**FIGURE 5:**
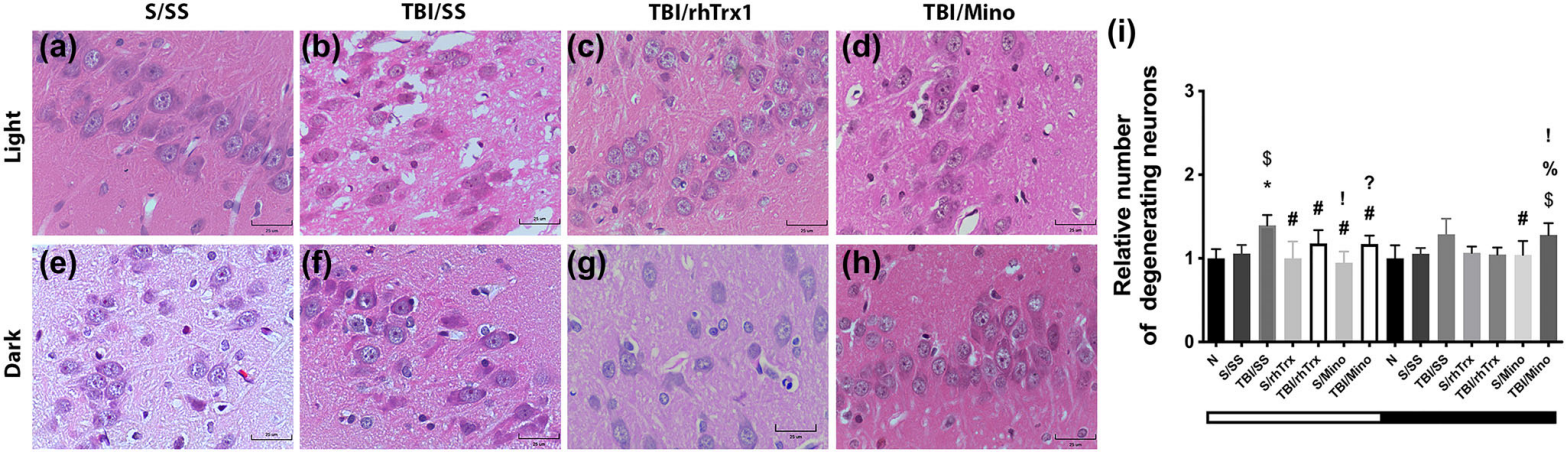
**The effect of rhTrx1 and minocycline on histopathology of the hippocampal subregion CA2/3**. Figures (a‐f) represent H&E staining of the hippocampal CA2/3 region in S/SS‐light phase (a), TBI/SS‐light phase (b), TBI/rhTrx1‐light phase (c), TBI/Mino‐light phase (d), S/SS‐dark phase (e), TBI/SS‐dark phase (f), TBI/rhTrx1‐dark phase (g), TBI/Mino‐dark phase (h) phase of the cycle, post‐TBI at 72 h. Relative number of degenerating neurons observed post‐TBI at 72 h with rhTrx1 and minocycline (i). In Light TBI/SS, we observed the loss of the typical histological arrangement of the CA2/3 region, mainly of the pyramidal cell layer (PCL) with degenerating neurons (cell body shrinkage, eosinophilic cytoplasm, and shrunken darkly stained (pyknotic nucleus), we also observed the loss of the integrity of the neuropil (b). For Light TBI/rhTrx1, we observed a modification in the PCL arrangement (c). In Light TBI/Mino, neuronal loss is notable, with changes due to acute eosinophilic neuron degeneration and a marked increase in the vascular lumen (d). In Dark TBI/SS, we found some cells with eosinophilia, but a preserved neuropil was found (f). In Dark TBI/rhTrx1, we observed a marked absence of changes suggestive of degeneration (g). Finally, in Dark TBI/Mino, some neurons show changes corresponding to acute eosinophilic neuron degeneration (h). Data are expressed as mean ± SEM (n = 4). Kruskal–Wallis and Dunn´s test as *post‐hoc*, *p < .05 versus Naïve (N), ^$^ *p* < .05 versus Sham (S), ^#^ *p* < .05 versus TBI/SS, ^%^ *p* < .05 versus S/rhTrx1, ^!^ *p* < .05 versus TBI/rhTrx, ^?^ *p* < .05 versus S/Mino. Groups S or TBI were administered with sterile saline (SS), thioredoxin (rhTrx), or minocycline (Mino) (bars, 25 μm).

Meanwhile, the relative number of degenerating neurons in hippocampal CA2/3 (Figure 5), the N group, was only significantly different from the TBI/SS group of the light phase. The S/SS group presented significant differences from TBI/SS (light phase) and TBI/Mino (dark phase) groups. TBI/SS group showed significant differences from all groups of the light phase and the S/Mino group of the dark phase. The S/rhTrx group was only significantly different from the TBI/Mino group of the dark phase. TBI/rhTrx group presented significant differences from S/Mino (light phase) and TBI/Mino (dark phase) groups. The S/Mino group showed only significant differences from the TBI/Mino group of the light phase.

**FIGURE 6:**
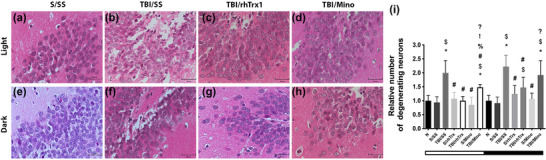
**The effect of rhTrx1 and minocycline on histopathology of the hippocampal subregion dentate gyrus (DG)**. Figures (a‐f) represent H&E staining of the hippocampal DG region in S/SS‐light phase (a), TBI/SS‐light phase (b), TBI/rhTrx1‐light phase (c), TBI/Mino‐light phase (d), S/SS‐dark phase (e), TBI/SS‐dark phase (f), TBI/rhTrx1‐dark phase (g), TBI/Mino‐dark phase (h) phase of the cycle, post‐TBI at 72 h. Relative number of degenerating neurons observed post‐TBI at 72 h with rhTrx1 and minocycline (i). In Light TBI/SS, we observed a loss of the granule cell layer (GCL) arrangement in the DG crest, with an abundant presence of acute eosinophilic neuron degeneration and loss of neuropil integrity (b). In Light TBI/rhTrx1, we observed preservation of the DG crest and the presence of some neurons with degeneration data (eosinophilic) (c); in fact, we observed a similar Light TBI/Mino pattern (d). In Dark TBI/SS, the DG crest area is also found with a loss of crest conformation in the GCL and with neurite degeneration data (f). However, in Dark TBI/rhTrx1, the area was more preserved, with little presence of degenerating neurons (g). Finally, in Dark TBI/Mino, the DG crest area is observed with a higher presence of eosinophilic neurons (h). Data are expressed as mean ± SEM (n = 4). Kruskal–Wallis and Dunn´s test as *post‐hoc*, *p < .05 versus Naïve (N), ^$^ *p* < .05 versus Sham (S), ^#^ *p* < .05 versus TBI/SS, ^%^ *p* < .05 versus S/rhTrx1, ^!^ *p* < .05 versus TBI/rhTrx, ^?^ *p* < .05 versus S/Mino. Groups S or TBI were administered with sterile saline (SS), thioredoxin (rhTrx), or minocycline (Mino) (bars, 25 μm).

The percentage of degenerating cells in DG was less (Figure 6), and the Naïve group significantly differed from the TBI/SS and TBI/Mino groups of both phases. The S/SS group presented significant differences from the TBI/SS and TBI/Mino groups of both phases and the TBI/rhTrx (dark phase) group. TBI/SS group showed significant differences from all groups, except for the TBI/Mino group of the dark phase. The S/rhTrx group was only significantly different from the TBI/Mino group of the light phase. TBI/rhTrx group presented only significant differences from the TBI/Mino group of the light phase. The S/Mino group showed significant differences from the TBI/Mino group of both phases. These data suggest less damage post‐TBI exists when experimental subjects are administered with Trx1 and minocycline, which is more notable if the injury occurs in the dark cycle.

**FIGURE 7:**
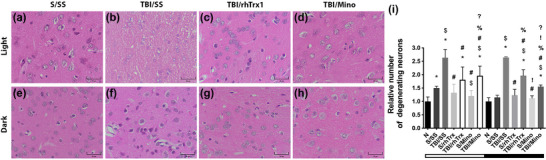
**The effect of rhTrx1 and minocycline on histopathology of the striatum**. Figures (a‐f) represent H&E staining of the dorsal striatum (caudate and putamen nucleus) region in S/SS‐light phase (a), TBI/SS‐light phase (b), TBI/rhTrx1‐light phase (c), TBI/Mino‐light phase (d), S/SS‐dark phase (e), TBI/SS‐dark phase (f), TBI/rhTrx1‐dark phase (g), TBI/Mino‐dark phase (h) phase of the cycle, post‐TBI at 72 h. Relative number of degenerating neurons observed post‐TBI at 72 h with rhTrx1 and minocycline (i). The striatum's histology (caudate‐putamen) comprises primarily medium‐sized neurons. In Light TBI/SS, we observed a loss in the number of neurons per field, with data suggestive of neuronal degeneration such as the presence of nuclei with karyorrhexis (b). In Light TBI/rhTrx1, neurons with basophilic changes and eosinophilic neurons were observed (c). In Light TBI/Mino, we also observed neuronal changes due to degeneration but in smaller amounts (d). In Dark TBI/SS, the presence of a large number of neurons with acute eosinophilic neuron degeneration is evident (f), while in Dark TBI/rhTRX1, we observed better preservation of the tissue, with a significant presence of medium‐sized neurons and few changes due to neuronal degeneration (g). Finally, in Dark TBI/Mino, we observed a retraction of neuronal size, with some neurons with pyknotic nuclei (h). Data are expressed as mean ± SEM (n = 4). Kruskal‐Wallis and Dunn´s test as *post‐hoc*, *p < .05 versus Naïve (N), ^$^ *p* < .05 versus Sham (S), ^#^ *p* < .05 versus TBI/SS, ^%^ *p* < .05 versus S/rhTrx1, ^!^ *p* < .05 versus TBI/rhTrx, ^?^ *p* < .05 versus S/Mino. Groups S or TBI were administered with sterile saline (SS), thioredoxin (rhTrx), or minocycline (Mino) (bars, 25 μm).

Finally, at 72 h post‐TBI in the dorsal striatum (Figure 7), the relative number of degenerating neurons in the Näive group significantly differed from S/SS, TBI/SS, TBI/rhTrx and TBI/Mino (light phase), and TBI/SS, TBI/Mino (dark phase) groups. The S/SS group presented significant differences from TBI/SS, S/Mino, TBI/Mino (light phases), and all TBI groups (dark phase). TBI/SS group showed significant differences from all groups. The S/rhTrx group significantly differed from TBI/Mino (both phases) and TBI/rhTrx (dark phase) groups. TBI/rhTrx group presented significant differences from S and TBI groups administered with Mino (dark phase). The S/Mino group showed only significant differences from TBI/Mino (both phases) group. No significant differences were observed between TBI/SS groups of light *v*ersus dark phases.

## 4 DISCUSSION

This study demonstrated the neuroprotective effect of intracortical administration of rhTrx1, which significantly reduced both motor and histopathological damage in the hippocampus and striatum regions, and pain in a murine model of mCCl. At the same time, we also observed that this effect depended on the phase of the light‐dark cycle during which TBI occurs.

Reducing body weight and food consumption is a metabolic reaction to stress (Sritharan & Thompson, 2009). After 24 h, all traumatized subjects showed a sudden drop in body weight and food intake, particularly noticeable in the rats subjected to TBI during the light cycle phase, followed by a rapid rebound three days later. However, neither rhTrx1 nor minocycline was able to alter this metabolic response, which might be attributed to the model utilized (mCCI), which despite its mildness, has the features of a mechanical and dynamic animal model whose influence is direct and enters the brain (Estrada‐Rojo et al., 2018a). Our research group previously found that recovery from TBI in a weight drop model in rats is improved if injury occurs during the dark phase of the cycle (Martinez‐Vargas et al., 2006), resulting in less body weight loss, and greater food intake when the impact occurs at 01:00 h rather than 13:00 h—suggesting that neuroprotection responses may have a diurnal variation. Furthermore, the loss in body weight and food intake was substantially different across groups in the light and dark phases of the cycle at 24 and 48 h after trauma, as well as a tendency to regain body weight and food intake post‐TBI (Martinez‐Tapia et al., 2020b). In another study, a drop in body weight was seen at 72 h and food intake at 24 and 72 h in rats treated to continuous light and under regular settings for 14 days, with a recovery of both parameters beginning on the seventh day (Li et al., 2016). However, Krishna et al. (2019), employing a fluid percussion injury model with similar features to CCl (Estrada‐Rojo et al., 2018a), found no significant changes in average daily food intake or decrease in body weight among the different groups of rats during 2 weeks of observation (Krishna et al., 2019).

In rats, damage to the sensorimotor cortex causes lasting neurological impairments that are most severe on the first day after TBI and subsequently recover (Sysoev et al., 2019). The balancing beam test (*beam walking*) can identify sensorimotor impairments in the forelimbs and hindlimbs of rats subjected to TBI and is used to evaluate the influence of treatment techniques on the recovery of motor behavior in models with cortical injury (Goldstein & Davis, 1990; Sysoev et al., 2019). In our study, when using the balance beam, motor recovery was observed 3 days after TBI, especially at 24 h post‐TBI when the rhTrx1 protein or minocycline was administered. We only obtained significant differences in the circular bar since walking through this beam is more complicated than the square bar (Gulinello, 2016). It has been observed that after a moderate CCI, the performance of rats on the balance beam decreases at 24 h, presenting a gradual recovery (Abrahamson et al., 2019; Bleimeister et al., 2019; Clark et al., 1997; Leary et al., 2017; Mountney et al., 2016; Okigbo et al., 2019; Radabugh et al., 2016; Shaw et al., 2013; Shear et al., 2016; Yan et al., 2000), even in mild CCI model (Yu et al., 2016). Furthermore, after a moderate CCI, significant differences in gross vestibulomotor function, finer components, and coordination by the beam balance task and beam walk task are obtained between traumatized and nontraumatized groups (Abrahamson et al., 2019; Bleimeister et al., 2019; Clark et al., 1997; Leary et al., 2017; Mountney et al., 2016; Okigbo et al., 2019; Radabaugh et al., 2016; Shaw et al., 2013; Shear et al., 2016; Yan et al., 2000). The effects of craniotomy had a minor but nonsignificant deleterious effect on walking delay in vehicle sham groups 24 h after TBI (Abrahamson et al., 2019; Bleimeister et al., 2019; Clark et al., 1997; Shear et al., 2016). In this work, no significant differences in motor improvement were detected between the traumatized groups in the day and night phases, probably related to the examination time. Li et al. (2016) discovered substantial latency changes in beam balancing and beam walking tests after 7 and 14 days of continuous light exposure, respectively, using a weight drop model (Li et al., 2016). According to our previous results, no differences in motor coordination across traumatized groups were identified either at 24 h or 8 days after a weight drop model (Martinez‐Vargas et al., 2006). It is critical to emphasize the favorable effect of rhTrx1 on motor recovery because no benefit or slight improvement was seen after TBI in various trials examining neuroprotective drugs (Dixon et al., 1999; Okigbo et al., 2019; Wang et al., 2014). In this sense, 24 h after TBI, rhTrx1 reduces motor impairment, while minocycline has no effect.

Many of TBI's usual sequelae, including depression, post‐traumatic stress disorder, and chronic pain, can be preceded, exacerbated, or perpetuated by insufficient or disrupted sleep (Wickwire et al., 2018). Acute and persistent pain is a typical side effect of a TBI. Acute pain is frequently linked with a definite tissue injury and lasts for many weeks before subsiding. On the other hand, chronic pain has a clear description, is persistent, and can last 3 to 6 months or more. It is also not as directly tied to tissue damage (Irvine *&* Clark, 2018). In this study, we evaluated spontaneous pain by quantifying the facial grimace used in some models of brain damage and trauma (Barahona et al., 2020; Saine et al., 2016; Shinozuka et al., 2020; Uddin et al., 2019). To a lesser degree, we observed a moderate pain response in subjects undergoing TBI during the dark phase of the cycle and with recovery 3 days after trauma.

In addition, there is a significant improvement in pain response in rats subjected to TBI in the light phase of the cycle and administered with rhTrx1 or minocycline. In a study of intracerebral hemorrhage (IH) and headache, as common consequences of a TBI, pain, and motor behavior were tested in rats, where a gradual recovery in the pain score was also detected using the RGS scale in the first days following the injury, as well as in the balance on the beam (Saine et al., 2016). After 3–5 weeks of trauma and early motor deficits, rats showed signs of persistent pain by presenting a higher score on the RGS scale compared to sham rats in a model of blast‐TBI, which is associated in humans with increased sensitivity to painful stimuli and chronic pain (Rosenfeld et al., 2013; Sayer, 2012; Tham et al., 2013). The pain response has circadian rhythms and fluctuates throughout the day (Bruguerolle & Labrecque, 2007; Chassard & Bruguerolle, 2004; Palada et al., 2020; Segal et al., 2018). Several rodent studies have also found that the dark phase of the cycle increases pain response capacity (Frederickson et al., 1977; Martínez‐Gómez et al., 1994; Oliverio et al., 1982).

Regarding structural damage, it has been reported that TBI causes damage and death of pyramidal neurons across Ammon's horn in rodents using the CCI model (Anderson et al., 2005; Colicos & Dash, 1996; McCullers et al., 2002). We observed an abundance of degenerating neurons (damaged and dead) in the DG subregion 72 h after TBI. This rate of damaged neurons was significantly lower when rhTrx1 was administered. In the CA1 subregion, the proportion of neurons in degeneration was more significant and more evident in rats subjected to TBI during the day phase of the cycle, which could be related to a high level of redox activity in these pyramidal neurons (Naseri Kouzehgarani et al., 2020); where interestingly, both rhTrx and minocycline reduced damage. In the CA2/3 subregion, there is less damage to CA1 and DG, which also decreases when rhTrx or minocycline is administered, but only in the rats subjected to TBI during the light phase of the cycle. According to our results, a study carried out in a model of lateral CCI plus hemorrhagic shock found a lower percentage of neuronal damage in CA3 compared to CA1. However, there were no significant changes between these subregions 7 days after the trauma (Exo et al., 2009). At 72 h post‐TBI in a lateral weight drop model, extremely substantial neuronal loss was seen in the hippocampus subregions CA1, CA2/3, and DG, with significant alterations in both CA1 and DG, compared to the dark phase of the cycle but not in CA2/3 (Martínez‐Tapia et al., 2020b). Several studies have found a greater susceptibility in the CA3 region than the CA1 region using the lateral CCI model within the first few days after TBI, possibly due to regional mechanical responses and cellular or physiological processes (Mao et al., 2013).

The dorsal striatum (caudate and putamen nucleus) is associated with voluntary motor processes; it initiates motor behavior, production, and sequencing (David et al., 2005). After chronic TBI, functional magnetic resonance imaging has revealed reduced caudate activity in response to a working memory task in patients with prefrontal cortex contusion (Wiese et al., 2006), in war veterans who have suffered a burst TBI (Newsome et al., 2015), and in children, where reduced functional connectivity between the motor network and voxels within the caudate is observed (Stephens et al., 2017). Moreover, this subcortical atrophy is linked to the microstructure of the white matter, implying that axonal lesions may contribute to subcortical volume loss (Leunissen et al., 2014). We discovered an abundance of neurons in degeneration in both phases of the cycle in the dorsal striatum 72 h after TBI, which diminishes in the presence of recombinant Trx1 and minocycline, which could be linked with motor behavior recovery. In rodents, axonal damage in the caudate–putamen has been identified following 24–72 h of TBI in a frontal impact + lateral rotation (Kilbourne et al., 2009) and in a bilateral mCCI model (Tong et al., 2002). In addition, 18 days after the TBI, using a bilateral mCCI model, the caudate–putamen region exhibits gliosis (astrocytes and microglia) both in the cellular areas and in the white fibers, and neurological tests reveal deficits in tongue mobility and transient deficits in forelimb positioning (Hoffman & Stein et al., 1994).

However, they do not report neuronal damage in these regions concerning diurnal variation. Circadian rhythms are highly regulated and have been maintained throughout evolution in various organisms, helping them to predict, prepare for, and adapt to environmental changes (Martínez‐Tapia et al., 2020a), as is the case with damage caused by a TBI. Our study confirms that there is less damage if the TBI occurs during hours of activity, which could be regardless of the type of injury (model used). In rodents with CNS injuries, less damage has been reported in the dark phase of the cycle (Beker et al., 2018; Schallner et al., 2017), and more significant damage or less recovery owing to circadian desynchronization due to the presence of dim or continuous light (Beker et al., 2018; Earnest et al., 2016; Fonken et al., 2019; Ramsey et al., 2020; Weil et al., 2020). There has been little research on TBI and its relationship to diurnal variation (Estrada‐Rojo et al., 2018b; Martinez‐Tapia et al., 2020b; Martinez‐Vargas et al., 2006, 2013) or circadian disruption (Li et al., 2016).

Because recombinant thioredoxin has been shown to have neuroprotective effects both in vitro (Bobba et al., 2014; Das et al., 1997; Lee et al., 2003; Zhang et al., 2018, 2012) and in vivo (Hattori et al., 2004; Tian et al., 2014; Wang et al., 2015; Zhang et al., 2009), some authors have proposed it as a prospective option for the treatment of ischemia, where it has been evaluated and all its benefits have been observed (Hattori et al., 2004; Jiao et al., 2020; Tian et al., 2014; Wang et al., 2015; Zhou et al., 2013). For example, rhTrx1 administration in vivo (middle cerebral artery occlusion) and in vitro (oxygen and glucose deprivation) reduces necroptosis, mitochondrial membrane potential damage, ROS accumulation, and NLRP3 inflammasome activation and favors the M2 phenotype over the M1, in addition to decreasing cerebral ischemic injury in mice by inhibiting the inflammatory response (Jiao et al., 2020). Thus, this is the first study to evaluate the neuroprotective effect of rhTrx1 in a TBI, detecting an improvement and early recovery of spontaneous pain, motor damage, and neuronal damage in the CA1, CA2/3, and DG subregions of the hippocampus. Baratz‐Goldstein et al. (2016) reported reduced cognitive damage caused by mild TBI in mice that received peptides derived from the Trx catalytic sequence (Baratz‐Goldstein et al., 2016). Furthermore, the Trx1 recombinant protein might be employed as an early preventative medicine in medical rehabilitation treatment following neurotrauma, both in hospitalized and outpatients, enhancing physical, sensory, and cognitive functions, which could aid the patient's quick recovery (Findlay et al., 2022). Trx1, for example, may offer otoprotection by inactivating apoptosis and lowering oxidative stress (Lu & Holmgren, 2014), processes that contribute to hearing loss caused by sonic trauma and electrode insertion (Mehkri et al., 2022).

## 5 CONCLUSIONS

Finally, although rats are nocturnal animals and humans are mainly diurnal, we consider that even with these differences, which we analyze taking our findings from rats to the human complex, there are interesting aspects to comment on that influence the mechanism of neuroprotection of diurnal variation. For example, melatonin may play an essential role as it presents a higher peak secretion during the dark phase (Bilu et al., 2016). Also, in a review, we have discussed the influence of the immune system, particularly microglia, and the specificity of the immune response in the CNS. For example, in the murine model, it has been observed that there are proinflammatory cytokines that decrease during the night but increase during the day, which promotes a lower proinflammatory microenvironment in the night phase (Guzman‐Ruiz et al., 2022; Martínez‐Tapia et al., 2020a); however, there are still few studies of this phenomenon in humans. Thus, our findings show that administering recombinant Trx1 reduces the damage induced by brain injury and confirms the diurnal variation of the damage caused by TBI. These findings imply that rhTrx1 might be a promising drug for treating TBI, even better than minocycline. Furthermore, diagnosis and therapy should consider a diurnal variation in damage and neuroprotection responses after TBI. As a result, it is critical to continue understanding the systems involved in these events to develop better therapies for optimal recovery after a TBI.

## CONFLICT OF INTEREST STATEMENT

The authors declare that they have no potential conflicts of interest concerning the work described.

### PEER REVIEW

The peer review history for this article is available at https://publons.com/publon/10.1002/brb3.3031.

## Data Availability

The data that support the findings of this study are available from the corresponding author upon reasonable request.
